# State-of-the-art colorectal disease: postoperative ileus

**DOI:** 10.1007/s00384-021-03939-1

**Published:** 2021-05-11

**Authors:** Nils P. Sommer, Reiner Schneider, Sven Wehner, Jörg C. Kalff, Tim O. Vilz

**Affiliations:** Department of Surgery, University Hospital Bonn, Bonn, Germany

**Keywords:** Postoperative ileus, Perioperative management, Prevention, Fast-track, Enhanced recovery

## Abstract

**Purpose:**

Postoperative Ileus (POI) remains an important complication for patients after abdominal surgery with an incidence of 10–27% representing an everyday issue for abdominal surgeons. It accounts for patients’ discomfort, increased morbidity, prolonged hospital stays, and a high economic burden. This review outlines the current understanding of POI pathophysiology and focuses on preventive treatments that have proven to be effective or at least show promising effects.

**Methods:**

Pathophysiology and recommendations for POI treatment are summarized on the basis of a selective literature review.

**Results:**

While a lot of therapies have been researched over the past decades, many of them failed to prove successful in meta-analyses. To date, there is no evidence-based treatment once POI has manifested. In the era of enhanced recovery after surgery or fast track regimes, a few approaches show a beneficial effect in preventing POI: multimodal, opioid-sparing analgesia with placement of epidural catheters or transverse abdominis plane block; μ-opioid-receptor antagonists; and goal-directed fluid therapy and in general the use of minimally invasive surgery.

**Conclusion:**

The results of different studies are often contradictory, as a concise definition of POI and reliable surrogate endpoints are still absent. These will be needed to advance POI research and provide clinicians with consistent data to improve the treatment strategies.

## Introduction

Postoperative ileus (POI) is a common problem encountered by surgeons after abdominal and even non-abdominal surgery [1]. Although physicians are quite familiar with this condition there is a lack of a precise clinical definition [2]. It is generally understood as a disruption of the regularly orchestrated, propulsive activity of the gastrointestinal tract after surgery. To a certain extent, this is considered as a normal, self-limited response to an operation. But sometimes prolonged paralysis occurs, leading to abdominal distension, nausea, and vomiting with the consequence of intolerance of oral food intake and delayed time to hospital discharge [3]. Multiple definitions have been used in the literature; most commonly, the absence of bowel movement and cessation of oral food intake longer than postoperative day 4 are the cutoff for prolonged POI [4]. But there is a broad variety in authors’ opinions about an adequate gastrointestinal recovery time. Additionally, prolonged POI must be differentiated from other complications such as early postoperative bowel obstruction, perforation, or intraabdominal abscess formation [5] which might present similarly but require a surgical intervention. As with the definition, the incidence of POI varies in surgical literature ranging from around 10% up to 27% of patients who are affected [6–8]. This does not only have a consequence for their wellbeing but is also related with higher morbidity and complications as well as a prolonged hospital stay and consecutively has a severe economic impact [5]. Recent data from New Zealand describe a significant increase by 71% of hospital costs in patients with prolonged ileus [9]. In the USA, the economic burden of POI was an estimated 750 million $ per year [10]. Therefore, POI remains a key issue for surgeons, patients, and society alike.

## Pathophysiology of POI

**Fig. 1:**
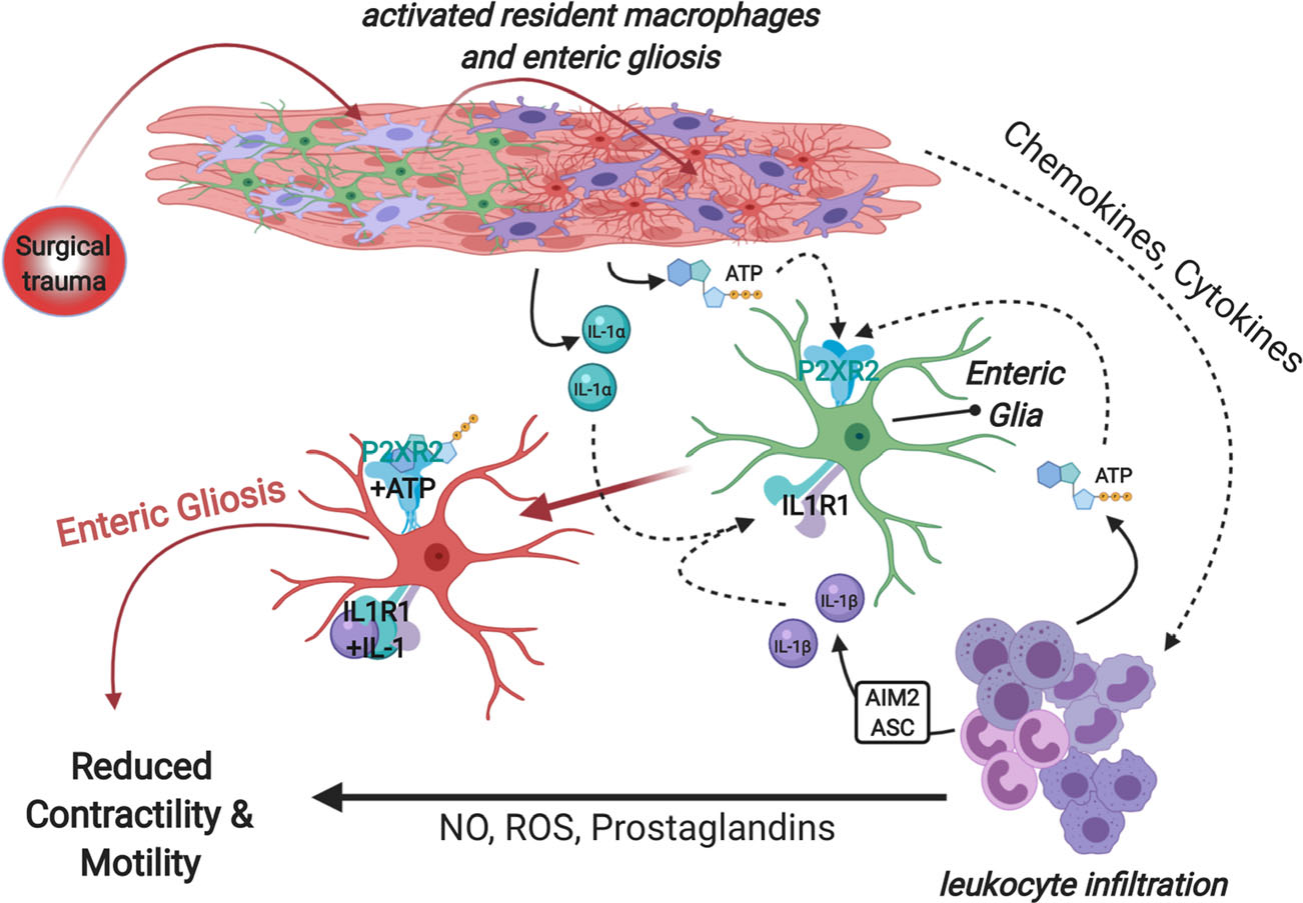
Current hypothesis of the complex orchestrated immunomodulatory effects on POI: Enteric glia are activated by purinoceptor (P2XR2) response to ATP and a IL-1 receptor type 1 (IL1R1) mediated response to IL-1α released after the trauma and IL-1ß that is dependent on the absent in melanoma 2 (AIM2) inflammasome. This and the activation of resident macrophages with consecutive leukocyte infiltration and release of nitric oxide (NO), reactive oxygen species (ROS), and prostaglandins contribute to POI development and provide potential new targets in POI prevention

The underlying mechanisms of POI are a complex interaction of inflammation, neural reflexes, neurohumoral pathways, and pharmacologic effects. While interstitial cells of cajal (ICC) provide the rhythmicity of gut motility by their pacemaker activity, the enteric nervous system is the key player in influencing gut motility with mediation by parasympathetic and sympathetic pathways [11, 12]. It has been demonstrated that manipulation of the gut leads to a leukocyte infiltration into the intestinal mucosa reflecting an inflammatory response which leads to an impaired muscle contractility [13, 14]. Further, animal studies revealed that a network of resident macrophages plays a central role in orchestrating this inflammation [15, 16] involving numerous cytokines but also prostaglandins by inducing the expression of cyclooxygenase-2 (COX-2) as well as NO [17]. Moreover, the release of proinflammatory cytokines and chemokines by the enteric nervous system add to that effect [18, 19]. Recently, research focused on the population of enteric glial cells. They modulate neural activity in the enteric nervous system and can be activated by mechanical forces that initiate a neuro-inflammatory process [20]. Preclinical research of our group demonstrated an IL-1 receptor-type 1 (IL1R1) and P2X2-dependend effect on POI [19]. Figure 1 provides a current understanding of the neuro-immune interactions in gastrointestinal dysmotility. Yet, not all mechanisms of action and their dependencies are fully understood. Furthermore, noxious stimuli of the surgical procedure trigger inhibitory neural reflexes by splanchnic afferents which are also mediated by supraspinal pathways, thus increasing sympathetic activity with reduced gut motility [11]. Aside from these noradrenergic facilitated effects, neurohumoral peptides such as nitric oxide (NO) and vasoactive intestinal polypeptide (VIP) also seem to act as inhibitory neurotransmitters leading to impaired intestinal motility [21]. Finally, the μ-receptor-mediated decrease of motility due to postoperative analgesia with opioids is a familiar contribution in maintaining POI [22].

## Risk factors for development of POI

The identification of predisposing factors associated with POI could allow for a targeted surveillance or even prophylaxis for patients at risk. Most meta-analyses searching for independent risk factors for POI have been published in a certain surgical field, commonly in patients undergoing colorectal surgery. The varying definition of POI hinders comparability of single studies and conclusions thereof [23]. For gastrointestinal surgery, male sex, creation of a stoma, respiratory comorbidities, and duration of surgery > 3 h were identified as independent risk factors as summarized in Table 1 [4, 23, 24]. One of the key factors contributing to POI is the surgical technique. It has been demonstrated that open surgery in contrast to minimally invasive surgery (MIS) significantly increases the probability of POI with odds ratios (OR) ranging from 1.97 to 6.37. Additionally, perioperative need for blood-transfusion and liberal crystalloid infusion contributes to POI [5, 7]. Predictive scores to identify the risk of POI have only been established in small- to medium-sized cohorts and have yet to be validated in clinical use [5, 25]. Furthermore, any factor (e.g., pharmacologic treatment, comorbidities) that reduces gastrointestinal motility per se can contribute to the risk for prolonged POI.

**Table 1:** Potential risk factors and their odds ratios (OR) for POI development. OR are provided according to the different studies cited; ranges do not represent 95% CI

Risk factor	Odds ratio (OR)
Male sex	1.4–1.8
Stoma creation	1.4–1.6
Surgery > 3 h	1.6–1.8
Open surgery	1.97–6.37
Respiratory comorbidities	1.11–1.9
Blood transfusion	1.8–2.0
Liberal crystalloid infusion regime (per liter)	1.55

## Diagnosis of POI

The main clinical features of POI are abdominal distension, nausea and vomiting, and the absence of flatus or stool passage. While there might be mild tenderness on palpation, usually, no muscular defense is present in clinical examination. Laboratory tests typically show no specific alterations [26, 27]. As postoperative complications like intra-abdominal infections and anastomotic leakage are also associated with POI, any patient with prolonged ileus should be closely monitored and these complications should always be ruled out [26].

## Prevention of POI

The risk factors collected over the years of POI research and the growing understanding of its pathophysiology have led to various prevention strategies. Those that have a promising effect on POI development are summarized in Table 2 and will be reviewed in this section. Some of them have been integrated in fast-track protocols. Their multimodal approach is to reduce or even prevent the undesirable side effects of patients’ pathophysiologic reactions to the surgical trauma and perioperative management [40]. Fast-track programs have gained popularity over the past two decades, yet their influence on POI has still to be investigated. Data suggest a beneficial effect on length of stay, time to defecation, and complications in patients treated under those regimens [41, 42] although it remains unclear which components account for the improvement.

**Table 2:** Summary of potential prevention strategies and their effect on aspects of POI. The quality of evidence and endpoints vary between the different studies and are discussed in the corresponding sections. (OR: odds ratio, SMD: standardized mean difference, LOS: length of hospital stay)

Prevention strategy	Effect
Peripheral μ-receptor antagonists	- Reduced incidence of POI (OR 0.67–0.77) [28] - Reduced LOS (OR 0.79 - 0.85) [29, 30]
Epidural catheter	- Reduced time to first flatus (SMD −1.14 to −1.28) or bowel movement (SMD −0.67 to −0.8) [31] - Reduced LOS for open surgery (SMD −0.2) [31]
TAP block	- Improved bowel function by 1 day [32] - Reduced LOS by 0.5–1 day [32, 33]
NSAIDs	- Reduced rate of POI after celecoxib (OR 0.1), no effect on recovery of bowel function [34]
Minimally-invasive surgery	- Reduced time to first flatus or bowel movement by 0.9 days [35] - Reduced LOS by 1–1.5 days [35, 36]
Chewing gum	- Reduced time to first flatus and bowel movement by 0.3–0.47 days [37]
Coffee consumption	- Reduced time to first defecation by 0.67 days, scarce data [38]
TENS	- Beneficial effect on gastrointestinal recovery, inconsistent data [39]

### Intraoperative and postoperative volume therapy

The infusion of fluids is supposed to compensate for the intraoperative fluid loss. Whether to opt for crystalloids or colloids is discussed widely, recently, no superiority was identified for either method [43, 44]. There are currently three regimes for fluid replacement, restrictive, liberal, and goal-directed volume therapies. Liberal protocols with traditional calculations (i.e., deficit: bodyweight + 40 kg × 1 ml/kg/h of fasting, maintenance: bodyweight + 40 kg × 1 ml/kg/h, third-space loss: 4–6 ml/kg/h) [45] might lead to edema of the gut wall which consecutively could result in POI. According to this hypothesis, it has been demonstrated that restrictive fluid management increases gastric emptying [46] which was also confirmed in a meta-analysis where liberal fluid administration was correlated to lengthier hospital stays and an increased time to bowel movement by 2 days [47]. Liberal protocols hence have been abandoned and replaced by restrictive or goal directed regimes. However, some trials associated restrictive fluid therapy with a higher rate of acute kidney injury compared to liberal regimes due to hypotension [48, 49]. Nonetheless, a recent meta-analysis showed a beneficial effect on perioperative complications for goal directed compared to restrictive volume therapy even though the certainty of the evidence was graded to be low. [50] Goal-directed fluid administration using transesophageally ultrasound is therefore part of many fast-track protocols. Yet, an ideal balance still has to be established to avoid the adverse effects of either too liberal or too strict protocols [40].

### Peripheral μ-receptor antagonists

Opioid use in postoperative analgesia leads to activation of central and peripheral opioid-receptors that facilitate their analgetic effect but their side effects as well. The μ-opioid receptor subtype located in the central nervous system is mainly responsible for analgesia, whilst peripheral μ-receptors mediate gastrointestinal dysfunction by inhibition of enteric nerve activity and propulsive motor activity. The development of μ-receptor specific antagonists (alvimopan and methylnaltrexone) that do not pass the blood-brain-barrier allowed for a specific inhibition of these side effects without affecting the analgesic potency [51]. Whilst alvimopan is approved by the US Food and Drug Administration (FDA) for the treatment of POI, methylnaltrexone is FDA-approved for therapy of opioid-induced constipation in a non-surgical setting only [52]. Alvimopan could demonstrate a reduction on the length of hospital stay by 0.62 days in a large propensity-matched cohort study of patients undergoing bowel resection [29]. This outcome was also verified in another database analysis of patients undergoing colorectal open surgery as well as MIS with a reduction by 1 day, respectively [30]. Yet, its role in MIS combined with fast-track protocols has still to be determined as there is controverse data showing no amelioration of POI [53]. Concerning the beneficial effect on the recovery of gastrointestinal function, a reduced incidence of POI was demonstrated [28] though not all studies found this to be of statistical significance [54, 55]. Methylnaltrexone, the other available μ-receptor antagonist, has also been investigated in phase II trials with promising effects on the burden of POI [56, 57]. Yet it failed to prove its efficacy in phase III trials concerning both gastrointestinal recovery and length of hospital stay [58].

### Opioid-sparing analgesia/multimodal analgesia

The opioid-mediated detrimental influence on POI led to opioid-sparing analgetic regimens. As a so-called multimodal analgesia, these protocols use a combination of different therapies (i.e., epidural catheter, transverse abdominis plane (TAP) block, NSAR), thus reducing opioid administration and have been implemented in various schemes into fast-track practices [40, 59].

#### Epidural catheter

The placement of an epidural catheter with continuous administration of a local anesthetic led to an accelerated recovery of gastrointestinal function as shown by a Cochrane review and its consecutive update [31, 60]. Aside from the opioid-sparing effect, the inhibition of visceral afferents and efferents that reduce intestinal motility is considered to be responsible. Therefore, thoracic placement is superior to lumbar epidurals [61]. However, the effectiveness of epidurals has to be investigated regarding MIS and fast track. A reduced length of stay was demonstrated for open surgery only but not for MIS [31]; furthermore, there is single-center acquired data suggesting no significant effect of epidurals on opioid consumption and length of stay after colorectal surgery regardless of the surgical technique which leads these authors to exclude epidural anesthesia from their fast-track protocols [62]. Considering the technical challenge of placing a thoracic epidural catheter and its side effects like urinary retention which might hinder patients’ mobilization, there is emerging use of alternative procedures.

#### Transverse abdominis plane (TAP) block

One of the most common alternatives to epidural catheters nowadays is the TAP block where a long-lasting local anesthetic (i.e., liposomal bupivacaine) is injected or a catheter is placed (for continuous application) between the internal oblique and transverse abdominis muscle layers guided by ultrasound providing a regional anesthesia [63]. As this technique is relatively new and was first introduced in 2001 [64], data regarding POI are sparse. There are results demonstrating reduced length of stay and opioid consumption as well as improved bowel function compared to traditional regimes after MIS [32]. Not many studies have compared TAP block to epidural catheters. While some authors showed reduced length of hospital stay with no difference in time to first flatus [33], others demonstrated lower ileus rates [63]. Regardless of the lack of meta-analyses, authors start to suggest implementation of TAP block into standard fast track protocols [65]. Yet more comparable studies are needed to prove the promising data.

#### Non-steroidal anti-inflammatory drugs (NSAIDs)

The role of COX-2 produced prostaglandins is known to play a central role in the development of POI in a murine model [17]. Therefore, the effect of NSAIDs has been investigated as it hypothetically could target a key step responsible for POI. In a randomized trial, treatment with celecoxib reduced the rate of paralytic ileus [34].

As the administration of COX-2 inhibitors also led to reduced opioid-doses and a faster recovery of patients [66, 67], the definitive course of action (either opioid-sparing effect or anti-inflammatory effect) remains to be determined. There has been growing evidence for the detrimental influence of diclofenac on anastomotic healing with higher leakage rates; consequently, COX-2 inhibitors should be used with caution [68]. Fortunately, this effect has not been verified for ketorolac [69] and ibuprofen [68], so their role in multimodal analgesia should not be impaired.

### Surgical approach

It is well known from research conducted in murine models on POI pathophysiology that the manipulation of the gut is directly associated with intestinal dysmotility. This effect was also verified in humans by the detection of tryptase and interleukin-(IL) 8 and 10 in the peritoneal fluid after open surgery which—aside from low levels of IL-8—was not seen after MIS [70]. It might therefore be an obvious conclusion to associate MIS with reduced POI. This hypothesis was supported by single-center trials [71] as well as meta-analyses [72, 73] demonstrating a beneficial effect on POI. A Cochrane review stated that patients undergoing MIS in colorectal surgery had faster flatus or bowel movement by 0.9-1 day compared to open surgery [35]. Furthermore, a prospective, multicentric trial conducted in the Netherlands on patients undergoing colonic surgery (LAFA-study) proved laparoscopy in combination with a fast-track program to be superior regarding morbidity and length of hospital stay compared to open surgery with or without a fast-track approach and MIS alone [36]. In a recent meta-analysis, patients had faster flatus after robotic right hemicolectomy compared to laparoscopic hemicolectomy, but no significant difference was observed for POI [74]. According to that, more research will be needed especially concerning MIS and robotic surgery in modern fast track programs.

### Additive options for POI prophylaxis

#### Chewing gum

The administration of chewing gum as a method of sham feeding has been established especially for patients who are intended to a delayed enteral feeding. Its effect is mediated by a cephalovagal reflex resulting in prokinetic parasympathomimetic activity [75]. The practice of gum chewing has been proven to be of statistical significance in POI (reduction in time to first bowel movement by 12.7 h) as mentioned in a 2015 Cochrane review. Although 81 studies have been included, the authors state the necessity for further randomized controlled trials as gum chewing regimens varied between studies and cohort sizes were mostly small [76]. Additionally, many fast-track protocols rely on an early oral food intake as a key procedure. In relation to that, the effect of a sham meal cannot be determined easily. In a more recent meta-analysis, the administration of chewing gum led to significant reduction of POI after colorectal surgery (RR 0.55) with improved time to flatus and defecation without affecting length of hospital stay [37]. Accordingly, gum chewing provides a safe and inexpensive intervention with a beneficial effect on POI so its routine use can be recommended.

#### Coffee consumption

A stimulating effect on bowel activity is contributed to coffee consumption in popular belief. Its mechanism of action is not well understood, but several pathways such as gastrin release, exorphins binding to opiate receptors, and the inhibition of adenosine receptors are hypothesized [77]. Recent meta-analyses demonstrated a significantly reduced time to first bowel movement after consuming coffee regularly in the postoperative period, but there was inconsistent data regarding its effect on length of hospital stay [38, 78]. This emphasizes that data on coffee and its role in POI prevention are scarce with only four and ten trials included in the meta-analyses mentioned above, respectively. Thus, more studies are needed to allow for a valid assessment. However, there seems to be no need to withhold coffee from patients tolerating a diet.

#### Transcutaneous electrical nerve stimulation/acupuncture

It has been hypothesized that neuromodulation can be used to promote gastrointestinal recovery according to its therapeutic uses in various diseases such as migraine and seizures. For instance, there is promising data that transcutaneous vagal stimulation reduces the inflammatory response within the intestinal wall and prevents POI in rodents via the activation of vagal efferents [79]. Also, electroacupuncture shortened gastrointestinal recovery time, yet no anti-inflammatory effect could be observed in animal studies [80]. Even though a prospective study proved electroacupuncture to reduce the duration of POI in humans after colorectal surgery about a decade ago [81], no larger cohort studies are available today. In a recent systematic review, a potentially beneficial effect of electrical stimulation (TENS, electroacupuncture, internal nerve stimulation) on POI was identified; nonetheless, heterogeneous study designs impede comparability of trials [39]. Furthermore, the direct mechanisms of action are not fully understood; therefore, the effect of neurostimulation on POI and its role in clinical regimens remains a good target for further investigation.

## Therapy of manifest POI

It has to be stated that once prolonged POI has manifested, there is no evidence-based therapeutic approach. Prokinetic substances like metoclopramide, erythromycin, and acetylcholinesterase-inhibitors such as neostigmine are widely used in clinical routine without showing a benefit in clinical symptoms or shortening POI in a Cochrane analysis [82]. Also, the oral administration of gastrografin, a hyperosmolar contrast agent, has no significant effect in resolving the symptoms of POI [83, 84]. Considering the multifactorial pathophysiology with a complex interaction of neuro-immune mechanisms and apparent inflammation of the muscularis externa, it is not surprising that a prokinetic agent fails to restore intestinal motility. The treatment of patients with manifest POI is of supportive manner only. This includes decompression of a distended intestine in patients with persistent vomiting via insertion of a nasogastric tube and parenteral nutrition depending on the duration of POI. Furthermore, isotonic crystalloids and potassium should be substituted intravenously to maintain normovolemia and balanced electrolytes [85].

## Conclusion and future directions

In summary, the incidence of POI remains high in patients even in the era of MIS and fast-track programs and is a burden for both patients and society. A Delphi approach within the Association of Coloproctology of Great Britain and Northern Ireland even stated POI to be in the high priority list of non-cancer related clinical problems [86]. A lot of research has been conducted regarding the pathophysiology and possible treatments. Current approaches focus either on advancing the understanding of promising prevention strategies, as we have discussed above or on new aspects of POI pathophysiology. Enteric glia and their IL1-receptor signaling pathways are an experimental target to influence neuroinflammation, yet clinical trials have not been initiated. In addition, prucalopride, a 5-HT4 receptor agonist reducing intestinal inflammation and vagus nerve stimulation, might provide treatment strategies if future studies demonstrate their safety and efficacy [20]. While there is certain evidence for single therapeutic options, these often fail to prove successful in meta-analyses. The reason lies within the studies itself. As stated in the introduction, there is no general definition of POI or prolonged POI which impedes comparability of acquired results due to varying endpoints. Some authors focused on that problem and tried to define parameters that best reflect restitution of gastrointestinal transit [87]. Also, a Delphi study found consensus to define POI [88]. Despite these efforts, the endpoints defined so far depend on soft criteria and often solely rely on patients’ compliance (e.g., time to first flatus, toleration of solid food). It is even unclear whether they are reliable criteria to assess the duration and severity of POI [89].

Efforts in POI research should focus on the development of reliable and, at its best, objective criteria allowing for qualitatively better studies and their comparability [90]. In our opinion, this is the only way to tackle the challenging clinical problem of POI.

## Data Availability

Not applicable

## References

[1] Bederman SS, Betsy M, Winiarsky R, Seldes RM, Sharrock NE, Sculco TP (2001). Postoperative ileus in the lower extremity arthroplasty patient. J Arthroplast.

[2] Vather R, Trivedi S, Bissett I (2013). Defining postoperative ileus: results of a systematic review and global survey. J Gastrointest Surg.

[3] Böhm B, Milsom JW, Fazio VW (1995). Postoperative intestinal motility following conventional and laparoscopic intestinal surgery. Arch Surg.

[4] Quiroga-Centeno AC, Jerez-Torra KA, Martin-Mojica PA, Castaneda-Alfonso SA, Castillo-Sanchez ME, Calvo-Corredor OF, Gomez-Ochoa SA (2020). Risk factors for prolonged postoperative ileus in colorectal surgery: a systematic review and meta-analysis. World J Surg.

[5] Moghadamyeghaneh Z, Hwang GS, Hanna MH, Phelan M, Carmichael JC, Mills S, Pigazzi A, Stamos MJ (2016). Risk factors for prolonged ileus following colon surgery. Surg Endosc.

[6] Iyer S, Saunders WB, Stemkowski S (2009). Economic burden of postoperative ileus associated with colectomy in the United States. J Manag Care Pharm.

[7] Vather R, Josephson R, Jaung R, Robertson J, Bissett I (2015). Development of a risk stratification system for the occurrence of prolonged postoperative ileus after colorectal surgery: a prospective risk factor analysis. Surgery.

[8] Wolthuis AM, Bislenghi G, Fieuws S, de Buck van Overstraeten A, Boeckxstaens G, D'Hoore A (2016). Incidence of prolonged postoperative ileus after colorectal surgery: a systematic review and meta-analysis. Color Dis.

[9] Mao H, Milne TGE, O'Grady G, Vather R, Edlin R, Bissett I (2019). Prolonged postoperative ileus significantly increases the cost of inpatient stay for patients undergoing elective colorectal surgery: results of a multivariate analysis of prospective data at a single institution. Dis Colon Rectum.

[10] Asgeirsson T, El-Badawi KI, Mahmood A, Barletta J, Luchtefeld M, Senagore AJ (2010). Postoperative ileus: it costs more than you expect. J Am Coll Surg.

[11] Boeckxstaens GE, de Jonge WJ (2009). Neuroimmune mechanisms in postoperative ileus. Gut.

[12] Takaki M (2003). Gut pacemaker cells: the interstitial cells of Cajal (ICC). J Smooth Muscle Res.

[13] Kalff JC, Schraut WH, Simmons RL, Bauer AJ (1998). Surgical manipulation of the gut elicits an intestinal muscularis inflammatory response resulting in postsurgical ileus. Ann Surg.

[14] Türler A, Moore BA, Pezzone MA, Overhaus M, Kalff JC, Bauer AJ (2002). Colonic postoperative inflammatory ileus in the rat. Ann Surg.

[15] Wehner S, Straesser S, Pantelis D, Sielecki T, de la Cruz VF, Hirner A, Kalff JC, Vilz TO (2009). Inhibition of p38 mitogen-activated protein kinase pathway as prophylaxis of postoperative ileus in mice. Gastroenterology.

[16] Wehner S, Sommer N, Sielecki T, Hong GS, Lysson M, Stoffels B, Pantelis D, Kalff JC, Vilz TO (2012). The novel orally active guanylhydrazone CPSI-2364 prevents postoperative ileus in mice independently of anti-inflammatory vagus nerve signaling. Langenbeck's Arch Surg.

[17] Schwarz NT, Kalff JC, Türler A, Engel BM, Watkins SC, Billiar TR, Bauer AJ (2001). Prostanoid production via COX-2 as a causative mechanism of rodent postoperative ileus. Gastroenterology.

[18] Stoffels B, Hupa KJ, Snoek SA, van Bree S, Stein K, Schwandt T, Lysson M, Veer CV, Kummer MP, Hornung V, Kalff JC, de Jonge WJ, Wehner S, Vilz TO (2014). Postoperative ileus involves interleukin-1 receptor signaling in enteric glia. Gastroenterology.

[19] Schneider R, Leven P, Glowka T, Kuzmanov I, Lysson M, Schneiker B, Miesen A, Baqi Y, Spanier C, Grants I, Mazzotta E, Villalobos-Hernandez E, Kalff JC, Müller CE, Christofi FL, Wehner S (2021). A novel P2X2-dependent purinergic mechanism of enteric gliosis in intestinal inflammation. EMBO Mol Med.

[20] Mazzotta E, Villalobos-Hernandez EC, Fiorda-Diaz J, Harzman A, Christofi FL (2020). Postoperative ileus and postoperative gastrointestinal tract dysfunction: pathogenic mechanisms and novel treatment strategies beyond colorectal enhanced recovery after surgery protocols. Front Pharmacol.

[21] Kalff JC, Schraut WH, Billiar TR, Simmons RL, Bauer AJ (2000). Role of inducible nitric oxide synthase in postoperative intestinal smooth muscle dysfunction in rodents. Gastroenterology.

[22] Holte K, Kehlet H (2000). Postoperative ileus: a preventable event. Br J Surg.

[23] Lee MJ, Vaughan-Shaw P, Vimalachandran D (2020). A systematic review and meta-analysis of baseline risk factors for the development of postoperative ileus in patients undergoing gastrointestinal surgery. Ann R Coll Surg Engl.

[24] Chapuis PH, Bokey L, Keshava A, Rickard MJ, Stewart P, Young CJ, Dent OF (2013). Risk factors for prolonged ileus after resection of colorectal cancer: an observational study of 2400 consecutive patients. Ann Surg.

[25] Kronberg U, Kiran RP, Soliman MS, Hammel JP, Galway U, Coffey JC, Fazio VW (2011). A characterization of factors determining postoperative ileus after laparoscopic colectomy enables the generation of a novel predictive score. Ann Surg.

[26] Stoffels B, Strassburg C, Schild HH, Kalff JC, Vilz TO (2017). Ileus in adults. Dtsch Arztebl Int.

[27] Wu Z, Boersema GS, Dereci A, Menon AG, Jeekel J, Lange JF (2015). Clinical endpoint, early detection, and differential diagnosis of postoperative ileus: a systematic review of the literature. Eur Surg Res.

[28] Al-Mazrou AM, Baser O, Kiran RP (2018). Alvimopan, Regardless of ileus risk, significantly impacts ileus, length of stay, and readmission after intestinal surgery. J Gastrointest Surg.

[29] Steele SR, Brady JT, Cao Z, Baumer DL, Robinson SB, Yang HK, Delaney CP (2018). Evaluation of healthcare use and clinical outcomes of alvimopan in patients undergoing bowel resection: a propensity score-matched analysis. Dis Colon Rectum.

[30] Henning RE, Hu KY, Rein LE, Szabo A, Peterson CY, Ludwig KA, Ridolfi TJ (2019). Alvimopan is associated with decreased length of stay for both open and laparoscopic segmental colectomy. Surgery.

[31] Guay J, Nishimori M, Kopp S (2016). Epidural local anaesthetics versus opioid-based analgesic regimens for postoperative gastrointestinal paralysis, vomiting and pain after abdominal surgery. Cochrane Database Syst Rev.

[32] Damadi AA, Lax EA, Smithson L, Pearlman RD (2019). Comparison of therapeutic benefit of bupivacaine HCl transversus abdominis plane (TAP) block as part of an enhanced recovery pathway versus traditional oral and intravenous pain control after minimally invasive colorectal surgery: a prospective, randomized, double-blind trial. Am Surg.

[33] Torgeson M, Kileny J, Pfeifer C, Narkiewicz L, Obi S (2018). Conventional epidural vs transversus abdominis plane block with liposomal bupivacaine: a randomized trial in colorectal surgery. J Am Coll Surg.

[34] Wattchow DA, De Fontgalland D, Bampton PA, Leach PL, McLaughlin K, Costa M (2009). Clinical trial: the impact of cyclooxygenase inhibitors on gastrointestinal recovery after major surgery—a randomized double blind controlled trial of celecoxib or diclofenac vs. placebo. Aliment Pharmacol Ther.

[35] Schwenk W, Haase O, Neudecker J, Müller JM (2005). Short term benefits for laparoscopic colorectal resection. Cochrane Database Syst Rev.

[36] Vlug MS, Wind J, Hollmann MW, Ubbink DT, Cense HA, Engel AF, Gerhards MF, van Wagensveld BA, van der Zaag ES, van Geloven AA, Sprangers MA, Cuesta MA, Bemelman WA, Group LS (2011). Laparoscopy in combination with fast track multimodal management is the best perioperative strategy in patients undergoing colonic surgery: a randomized clinical trial (LAFA-study). Ann Surg.

[37] Roslan F, Kushairi A, Cappuyns L, Daliya P, Adiamah A (2020). The impact of sham feeding with chewing gum on postoperative ileus following colorectal surgery: a meta-analysis of randomised controlled trials. J Gastrointest Surg.

[38] Gkegkes ID, Minis EE, Iavazzo C (2020). Effect of caffeine intake on postoperative ileus: a systematic review and meta-analysis. Dig Surg.

[39] Penfold JA, Wells CI, Du P, Bissett IP, O'Grady G (2019). Electrical stimulation and recovery of gastrointestinal function following surgery: a systematic review. Neuromodulation.

[40] Kehlet H (2020). Enhanced postoperative recovery: good from afar, but far from good?. Anaesthesia.

[41] Basse L, Thorbøl JE, Løssl K, Kehlet H (2004). Colonic surgery with accelerated rehabilitation or conventional care. Dis Colon Rectum.

[42] Muller S, Zalunardo MP, Hubner M, Clavien PA, Demartines N (2009). A fast-track program reduces complications and length of hospital stay after open colonic surgery. Gastroenterology.

[43] Lewis SR, Pritchard MW, Evans DJ, Butler AR, Alderson P, Smith AF, Roberts I (2018). Colloids versus crystalloids for fluid resuscitation in critically ill people. Cochrane Database Syst Rev.

[44] Kabon B, Sessler DI, Kurz A (2019). Effect of intraoperative goal-directed balanced crystalloid versus colloid administration on major postoperative morbidity: a randomized trial. Anesthesiology.

[45] Boland MR, Noorani A, Varty K, Coffey JC, Agha R, Walsh SR (2013). Perioperative fluid restriction in major abdominal surgery: systematic review and meta-analysis of randomized, clinical trials. World J Surg.

[46] Lobo DN, Bostock KA, Neal KR, Perkins AC, Rowlands BJ, Allison SP (2002). Effect of salt and water balance on recovery of gastrointestinal function after elective colonic resection: a randomised controlled trial. Lancet.

[47] Corcoran T, Rhodes JE, Clarke S, Myles PS, Ho KM (2012). Perioperative fluid management strategies in major surgery: a stratified meta-analysis. Anesth Analg.

[48] Myles PS, Bellomo R, Corcoran T, Forbes A, Peyton P, Story D, Christophi C, Leslie K, McGuinness S, Parke R, Serpell J, Chan MTV, Painter T, McCluskey S, Minto G, Wallace S (2018). Restrictive versus liberal fluid therapy for major abdominal surgery. N Engl J Med.

[49] Furrer MA, Schneider MP, Löffel LM, Burkhard FC, Wuethrich PY (2018). Impact of intra-operative fluid and noradrenaline administration on early postoperative renal function after cystectomy and urinary diversion: a retrospective observational cohort study. Eur J Anaesthesiol.

[50] Wrzosek A, Jakowicka-Wordliczek J, Zajaczkowska R, Serednicki WT, Jankowski M, Bala MM, Swierz MJ, Polak M, Wordliczek J (2019). Perioperative restrictive versus goal-directed fluid therapy for adults undergoing major non-cardiac surgery. Cochrane Database Syst Rev.

[51] Becker G, Blum HE (2009). Novel opioid antagonists for opioid-induced bowel dysfunction and postoperative ileus. Lancet.

[52] Chamie K, Golla V, Lenis AT, Lec PM, Rahman S, Viscusi ER (2020). Peripherally acting mu-opioid receptor antagonists in the management of postoperative ileus: a clinical review. J Gastrointest Surg.

[53] Keller DS, Flores-Gonzalez JR, Ibarra S, Mahmood A, Haas EM (2016). Is there value in alvimopan in minimally invasive colorectal surgery?. Am J Surg.

[54] Nemeth ZH, Bogdanovski DA, Paglinco SR, Barratt-Stopper P, Rolandelli RH (2017). Cost and efficacy examination of alvimopan for the prevention of postoperative ileus. J Investig Med.

[55] Buchler MW, Seiler CM, Monson JR, Flamant Y, Thompson-Fawcett MW, Byrne MM, Mortensen ER, Altman JF, Williamson R (2008). Clinical trial: alvimopan for the management of post-operative ileus after abdominal surgery: results of an international randomized, double-blind, multicentre, placebo-controlled clinical study. Aliment Pharmacol Ther.

[56] Anissian L, Schwartz HW, Vincent K, Vincent HK, Carpenito J, Stambler N, Ramakrishna T (2012). Subcutaneous methylnaltrexone for treatment of acute opioid-induced constipation: phase 2 study in rehabilitation after orthopedic surgery. J Hosp Med.

[57] Viscusi ER, Rathmell JP, Fichera A, Binderow SR, Israel RJ, Galasso FL, Penenberg D, Gan TJ (2013). Randomized placebo-controlled study of intravenous methylnaltrexone in postoperative ileus. J Drug Assess.

[58] Yu CS, Chun HK, Stambler N, Carpenito J, Schulman S, Tzanis E, Randazzo B (2011). Safety and efficacy of methylnaltrexone in shortening the duration of postoperative ileus following segmental colectomy: results of two randomized, placebo-controlled phase 3 trials. Dis Colon Rectum.

[59] Joshi GP, Kehlet H, Group PW (2017). Guidelines for perioperative pain management: need for re-evaluation. Br J Anaesth.

[60] Jorgensen H, Wetterslev J, Moiniche S, Dahl JB (2000). Epidural local anaesthetics versus opioid-based analgesic regimens on postoperative gastrointestinal paralysis, PONV and pain after abdominal surgery. Cochrane Database Syst Rev.

[61] Steinbrook RA (1998). Epidural anesthesia and gastrointestinal motility. Anesth Analg.

[62] Rosen DR, Wolfe RC, Damle A, Atallah C, Chapman WC, Vetter JM, Mutch MG, Hunt SR, Glasgow SC, Wise PE, Smith RK, Silviera ML (2018). Thoracic epidural analgesia: does it enhance recovery?. Dis Colon Rectum.

[63] Pirrera B, Alagna V, Lucchi A, Berti P, Gabbianelli C, Martorelli G, Mozzoni L, Ruggeri F, Ingardia A, Nardi G, Garulli G (2018). Transversus abdominis plane (TAP) block versus thoracic epidural analgesia (TEA) in laparoscopic colon surgery in the ERAS program. Surg Endosc.

[64] Rafi AN (2001). Abdominal field block: a new approach via the lumbar triangle. Anaesthesia.

[65] Fields AC, Weiner SG, Maldonado LJ, Cavallaro PM, Melnitchouk N, Goldberg J, Stopfkuchen-Evans MF, Baker O, Bordeianou LG, Bleday R (2020). Implementation of liposomal bupivacaine transversus abdominis plane blocks into the colorectal enhanced recovery after surgery protocol: a natural experiment. Int J Color Dis.

[66] Chen JY, Ko TL, Wen YR, Wu SC, Chou YH, Yien HW, Kuo CD (2009). Opioid-sparing effects of ketorolac and its correlation with the recovery of postoperative bowel function in colorectal surgery patients: a prospective randomized double-blinded study. Clin J Pain.

[67] Sim R, Cheong DM, Wong KS, Lee BM, Liew QY (2007). Prospective randomized, double-blind, placebo-controlled study of pre- and postoperative administration of a COX-2-specific inhibitor as opioid-sparing analgesia in major colorectal surgery. Color Dis.

[68] Klein M, Gögenur I, Rosenberg J (2012). Postoperative use of non-steroidal anti-inflammatory drugs in patients with anastomotic leakage requiring reoperation after colorectal resection: cohort study based on prospective data. Bmj.

[69] Hawkins AT, McEvoy MD, Wanderer JP, Ford MM, Hopkins MB, Muldoon RL, Martin BJ, King AB, Geiger TM (2018). Ketorolac use and anastomotic leak in elective colorectal surgery: a detailed analysis. Dis Colon Rectum.

[70] Bennink RJ, Ankum WM, Buist MR, Busch OR, Gouma DJ, van der Heide S, van den Wijngaard RM, de Jonge WJ, Boeckxstaens GE, The FO (2008). Intestinal handling-induced mast cell activation and inflammation in human postoperative ileus. Gut.

[71] Schwenk W, Böhm B, Haase O, Junghans T, Müller JM (1998). Laparoscopic versus conventional colorectal resection: a prospective randomised study of postoperative ileus and early postoperative feeding. Langenbeck's Arch Surg.

[72] Abraham NS, Young JM, Solomon MJ (2004). Meta-analysis of short-term outcomes after laparoscopic resection for colorectal cancer. Br J Surg.

[73] Ohtani H, Tamamori Y, Arimoto Y, Nishiguchi Y, Maeda K, Hirakawa K (2012). A meta-analysis of the short- and long-term results of randomized controlled trials that compared laparoscopy-assisted and open colectomy for colon cancer. J Cancer.

[74] Genova P, Pantuso G, Cipolla C, Latteri MA, Abdalla S, Paquet JC, Brunetti F, De’Angelis N, Di Saverio S (2020) Laparoscopic versus robotic right colectomy with extra-corporeal or intra-corporeal anastomosis: a systematic review and meta-analysis. Langenbecks Arch Surg 10.1007/s00423-020-01985-x 10.1007/s00423-020-01985-x 32902707

[75] Su'a BU, Pollock TT, Lemanu DP, MacCormick AD, Connolly AB, Hill AG (2015). Chewing gum and postoperative ileus in adults: a systematic literature review and meta-analysis. Int J Surg.

[76] Short V, Herbert G, Perry R, Atkinson C, Ness AR, Penfold C, Thomas S, Andersen HK, Lewis SJ (2015). Chewing gum for postoperative recovery of gastrointestinal function. Cochrane Database Syst Rev.

[77] Gungorduk K, Paskal EK, Demirayak G, Köseoğlu SB, Akbaba E, Ozdemir IA (2020). Coffee consumption for recovery of intestinal function after laparoscopic gynecological surgery: A randomized controlled trial. Int J Surg.

[78] Hogan S, Steffens D, Rangan A, Solomon M, Carey S (2019). The effect of diets delivered into the gastrointestinal tract on gut motility after colorectal surgery-a systematic review and meta-analysis of randomised controlled trials. Eur J Clin Nutr.

[79] Hong GS, Zillekens A, Schneiker B, Pantelis D, de Jonge WJ, Schaefer N, Kalff JC, Wehner S (2019). Non-invasive transcutaneous auricular vagus nerve stimulation prevents postoperative ileus and endotoxemia in mice. Neurogastroenterol Motil.

[80] Fang JF, Fang JQ, Shao XM, Du JY, Liang Y, Wang W, Liu Z (2017). Electroacupuncture treatment partly promotes the recovery time of postoperative ileus by activating the vagus nerve but not regulating local inflammation. Sci Rep.

[81] Ng SS, Leung WW, Mak TW, Hon SS, Li JC, Wong CY, Tsoi KK, Lee JF (2013). Electroacupuncture reduces duration of postoperative ileus after laparoscopic surgery for colorectal cancer. Gastroenterology.

[82] Traut U, Brügger L, Kunz R, Pauli-Magnus C, Haug K, Bucher HC, Koller MT (2008). Systemic prokinetic pharmacologic treatment for postoperative adynamic ileus following abdominal surgery in adults. Cochrane Database Syst Rev.

[83] Vather R, Josephson R, Jaung R, Kahokehr A, Sammour T, Bissett I (2015). Gastrografin in prolonged postoperative ileus: a double-blinded randomized controlled trial. Ann Surg.

[84] Biondo S, Miquel J, Espin-Basany E, Sanchez JL, Golda T, Ferrer-Artola AM, Codina-Cazador A, Frago R, Kreisler E (2016). A double-blinded randomized clinical study on the therapeutic effect of gastrografin in prolonged postoperative ileus after elective colorectal surgery. World J Surg.

[85] Vather R, Bissett I (2013). Management of prolonged post-operative ileus: evidence-based recommendations. ANZ J Surg.

[86] Tiernan J, Cook A, Geh I, George B, Magill L, Northover J, Verjee A, Wheeler J, Fearnhead N (2014). Use of a modified Delphi approach to develop research priorities for the association of coloproctology of Great Britain and Ireland. Color Dis.

[87] van Bree SH, Bemelman WA, Hollmann MW, Zwinderman AH, Matteoli G, El Temna S, Vlug MS, Bennink RJ, Boeckxstaens GE, The FO (2014). Identification of clinical outcome measures for recovery of gastrointestinal motility in postoperative ileus. Ann Surg.

[88] Gero D, Gie O, Hubner M, Demartines N, Hahnloser D (2017). Postoperative ileus: in search of an international consensus on definition, diagnosis, and treatment. Langenbeck's Arch Surg.

[89] Roessel L, Chang J, Pantelis D, Schwandt T, Koscielny A, Wehner S, Kalff JC, Vilz TO (2015). Establishing a biomarker for postoperative ileus in humans—results of the BiPOI trial. Life Sci.

[90] Pantelis D, Lingohr P, Fimmers R, Esmann A, Randau T, Kalff JC, Coenen M, Wehner S, Vilz TO (2016). SmartPill® as an objective parameter for determination of severity and duration of postoperative ileus: study protocol of a prospective, two-arm, open-label trial (the PIDuSA study). BMJ Open.

